# GAMT-GINE: A Graph Isomorphism Network Integrating Continuous Spatial Awareness and Multi-Task Learning for Protein–Ligand Binding Affinity Prediction

**DOI:** 10.3390/ijms27167196

**Published:** 2026-08-12

**Authors:** Jiarui Li, Hongquan Li, Di Wu, Wei He, Weinan Cao, Hua Yang, Zhen Hou

**Affiliations:** 1 School of Information Science and Engineering, Shanxi Agricultural University, Jinzhong 030801, China; 20230302420@stu.sxau.edu.cn (J.L.); 20221210616@stu.sxau.edu.cn (D.W.); hewei2@sxau.edu.cn (W.H.); caoweinan@sxau.edu.cn (W.C.); 2 Shanxi Key Lab for Modernization of TCVM, College of Veterinary Medicine, Shanxi Agricultural University, Jinzhong 030801, China; livets@163.com

**Keywords:** protein–ligand affinity, deep learning, graph isomorphism network, multi-task learning, continuous spatial perception

## Abstract

Protein–ligand interactions (PLIs) play a crucial role in drug discovery, and accurately predicting protein–ligand binding affinity (PLA) remains a central challenge in computer-aided drug design. Although graph neural networks (GNNs) have demonstrated considerable potential in molecular modeling, existing methods still face several limitations, including excessive reliance on hand-crafted chemical features, loss of spatial information, and difficulties in integrating heterogeneous affinity labels, which restrict their generalization capability in PLA prediction. To address these challenges, we propose GAMT-GINE, a graph isomorphism network that integrates continuous spatial awareness with multi-task learning. The model employs minimalist atomic features and a batch-normalization-free mechanism, together with a multi-task branch that uses a large amount of half-maximal inhibitory concentration (IC_50_) data as an auxiliary prediction target. Experimental results show that GAMT-GINE achieves a Pearson’s correlation coefficient (*R*_p_) of 0.791 and a root mean square error (RMSE) of 1.403 on the CASF-2013 benchmark dataset. In the generalization evaluation on CASF-2016, *R*_p_ further increases to 0.831, while RMSE decreases to 1.227, demonstrating performance comparable to that of current State-of-the-Art models. Furthermore, comprehensive evaluations, including ablation studies, feature importance analysis, analysis of the effects of data filtering on model performance and data composition, and analysis of the influence of training–test data similarity on prediction results, indicate that GAMT-GINE can effectively utilize continuous spatial information and heterogeneous affinity labels, achieving good predictive accuracy and cross-dataset generalization capability.

## 1. Introduction

Drug discovery has long faced major challenges, including lengthy development timelines, high costs, and limited success rates of candidate compounds [1,2]. The rapid expansion of accessible chemical space has further increased the difficulty of early-stage drug screening [3]. Against this background, computer-aided drug design has become an important approach for improving the efficiency of candidate compound screening and reducing research and development costs [4,5]. In particular, accurate prediction of protein–ligand binding affinity (PLA) facilitates the evaluation of binding strength between candidate compounds and their targets, enables the efficient identification of promising hit compounds, and accelerates lead discovery and optimization [6,7]. However, protein–ligand binding is a highly complex and dynamic physicochemical process at the molecular scale. It involves multiple types of non-covalent interactions and depends strongly on the precise three-dimensional conformation of the target binding pocket. Consequently, accurate PLA prediction remains a considerable challenge [8,9].

Experimental characterization of protein–ligand binding has traditionally relied on X-ray crystallography [10], nuclear magnetic resonance (NMR) spectroscopy [11], and other related biophysical techniques. Although these experimental approaches can provide highly reliable structural and interaction information, they generally require stringent experimental conditions, relatively long experimental cycles, and substantial financial resources. They are therefore difficult to apply directly to the high-throughput screening of large compound libraries during early-stage drug discovery [12,13]. Consequently, computer-aided drug design [14] has increasingly become an important means of accelerating the screening of early-stage candidate compounds. Conventional computational methods, such as molecular docking [15] and molecular dynamics simulations [16], have substantially improved screening efficiency. However, they still face challenges in achieving an optimal balance between computational cost and predictive accuracy. Owing to its capacity to process complex, high-dimensional data and learn nonlinear feature representations, deep learning has provided a new technological route for accurate PLA prediction [17]. In particular, advances in graph neural networks (GNNs) have enabled researchers to represent protein–ligand complexes as graph-structured data and directly predict binding affinity in an end-to-end manner, thereby accelerating progress in this field [18].

Among existing affinity prediction methods, some early approaches to three-dimensional structural representation adopted spatial discretization strategies. For example, Pafnucy [19] represents protein–ligand complexes using three-dimensional voxel grids, whereas OnionNet [20] describes intermolecular interactions through multiple distance shells. However, such discrete spatial partitioning strategies may fail to adequately preserve the smooth variation in continuous interatomic distances. Moreover, these methods rely heavily on complex chemical features, such as hybridization states, implicit valences, and ring-membership indicators, which may limit the ability of neural networks to autonomously learn fundamental physicochemical patterns from molecular structures. To address these limitations, subsequent studies such as InteractionGraphNet (IGN) [21] introduced graph neural networks to capture intermolecular topological relationships. Nevertheless, graph construction in these models still depends on fixed distance cutoffs and has not completely eliminated reliance on hand-crafted chemical features. An examination of recent graph neural network models and three-dimensional structure-based models, including FAST [22], suggests that conventional batch normalization during deep message passing may attenuate intrinsic variations in absolute spatial features across molecules [23], thereby weakening the representation of fine-grained three-dimensional geometric relationships. In addition, most existing models are trained in a single-task setting using strictly filtered, high-quality datasets. On the one hand, directly excluding large numbers of samples derived from NMR structures, low-resolution structures, or affinity measurements with ambiguous boundaries may reduce the structural diversity of the training data and limit model robustness to noisy and heterogeneous samples [24]. On the other hand, although precise affinity labels such as dissociation constants (*K*_d_) and inhibition constants (*K*_i_) constitute a major proportion of real-world biochemical databases, a considerable number of high-quality three-dimensional structures are associated only with alternative assay measurements, such as half-maximal inhibitory concentrations (IC_50_) [25]. Conventional single-task models have difficulty integrating heterogeneous affinity labels and therefore often discard these samples, resulting in the loss of valuable structural and chemical-space information.

To address the aforementioned limitations, this study proposes GAMT-GINE, a graph isomorphism network that integrates continuous spatial awareness with multi-task learning. Graph isomorphism networks are a class of graph neural networks that learn local graph structural features by aggregating information from neighboring nodes and their connections. In GAMT-GINE, a protein–ligand complex is represented as a graph composed of atoms and their spatial contact relationships, where atoms are treated as nodes and edges are established between spatially proximal protein and ligand atoms. In this representation, node features describe atom types, whereas edge features characterize the three-dimensional distances between neighboring atoms. Based on this graph representation, GAMT-GINE employs 17-dimensional basic atomic features to encode different atom types and uses radial basis functions (RBFs) [26] to encode continuous interatomic distances. RBF encoding transforms a single distance value into a set of smoothly varying features, enabling the model to capture subtle spatial distance variations at the protein–ligand interface. The model also removes conventional batch normalization to reduce the potential influence of feature standardization on the scale of spatial information across different complexes. In addition, to make use of heterogeneous experimental affinity data, this study adopts a multi-task learning strategy that combines a primary task with an auxiliary task, in which *K*_d_ and *K*_i_ data are used for the primary affinity prediction task, whereas IC_50_ data are used as an auxiliary task to provide additional training information. The two tasks share the same feature-extraction module, allowing samples with only IC_50_ labels to contribute to the learning of protein–ligand structural features. Finally, the model is trained using datasets including PDBbind v2020.R1 [27] and evaluated on independent CASF benchmark datasets.

## 2. Results and Discussion

This section first presents statistical analyses of the affinity-label composition, pK distributions, experimental structure-determination methods, and crystallographic resolutions of the training and CASF benchmark datasets, thereby establishing the data basis for subsequent model training and evaluation. Subsequently, the performance of GAMT-GINE in protein–ligand binding affinity (PLA) prediction is evaluated using three metrics: root mean square error (RMSE), mean absolute error (MAE), and the Pearson correlation coefficient (Rp), and is compared with existing methods on the CASF-2013 [28] and CASF-2016 [29] benchmark test sets. Ablation studies examine the contributions of the key model components to predictive performance, whereas feature importance analysis further investigates the effects of different input features on model outputs. In addition, model performance under different structural and affinity-label filtering strategies is compared, and the effects of these filtering procedures on training-set size, affinity distributions, and target structural composition are analyzed to clarify the relationship between data filtering and changes in external test performance. Finally, this study further investigated the relationship between training–test data similarity and model prediction performance and discussed the relationship of GAMT-GINE with emerging biomolecular structure prediction methods, such as AlphaFold 3 and RoseTTAFold All-Atom, in terms of task objectives and potential application workflows. Building on this analysis, a retrospective multi-target candidate prioritization analysis was conducted using the CASF-2016 test set, with PDE10A selected as a representative case to further evaluate the potential utility of GAMT-GINE for candidate prioritization.

### 2.1. Dataset Composition and Characteristics

**Figure 1:**
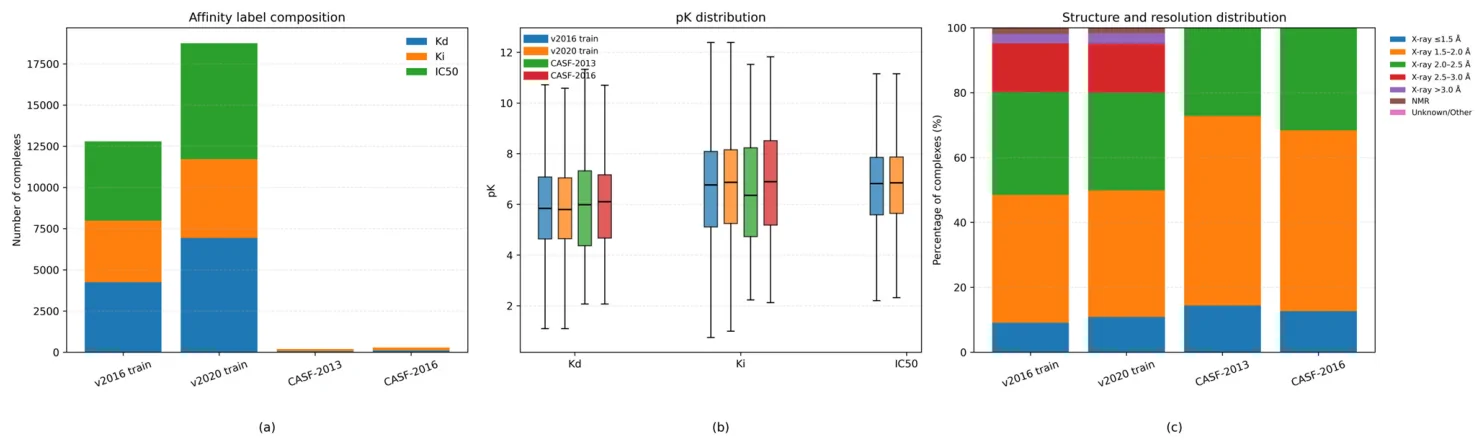
Composition and distribution of the protein–ligand datasets. (**a**) Numbers of samples labeled with *K*_d_, *K*_i_, and IC_50_ in each dataset; (**b**) *pK* distributions across different datasets and affinity types; and (**c**) proportional distributions of different experimental structure-determination methods and crystallographic resolution ranges. In the box plots, the central line represents the median, the lower and upper boundaries of each box represent the first and third quartiles, respectively, and the whiskers extend to the most extreme observations within 1.5 times the interquartile range.

The composition and binding-affinity statistics of the v2016 and v2020.R1 training sets and the CASF-2013 and CASF-2016 independent test sets are summarized in Table 1. In the table, Total N denotes the number of protein–ligand complexes in each dataset, *pK* range represents the minimum and maximum reference *pK* values, and *pK* median (IQR) represents the median reference *pK* value and interquartile range, with the IQR calculated as the difference between the third and first quartiles. The v2016 and v2020.R1 training sets contained 12,795 and 18,752 protein–ligand complexes, respectively. The v2016 training set comprised 4248 *K*_d_ samples, 3736 *K*_i_ samples, and 4811 IC_50_ samples, whereas the corresponding numbers in the v2020.R1 training set were 6936, 4783, and 7033, respectively. In contrast, CASF-2013 and CASF-2016 contained only *K*_d_ and *K*_i_ labels, with no IC_50_ samples. Specifically, 130 samples in CASF-2013 were labeled with *K*_i_, accounting for 66.67% of the dataset, whereas 178 samples in CASF-2016 were labeled with *K*_i_, accounting for 62.46% (Figure 1a).

To facilitate comparison of affinity values spanning multiple orders of magnitude, experimental measurements were expressed on the *pK* scale; an increase of 1 *pK* unit corresponds to a tenfold decrease in the original molar concentration. The overall median *pK* values of the v2016 and v2020.R1 training sets were 6.47 and 6.46, respectively, with corresponding interquartile ranges of 2.64 and 2.60. Both training sets had a *pK* range of 0.40–15.22. The overall median *pK* values of CASF-2013 and CASF-2016 were 6.27 and 6.50, respectively, with interquartile ranges of 3.38 and 3.12 and value ranges of 2.07–11.52 and 2.07–11.82, respectively (Table 1). Comparison among affinity-label types showed that the *pK* distributions of the *K*_i_ and IC_50_ samples generally tended to be higher than those of the *K*_d_ samples. Nevertheless, substantial overlap remained among different affinity types and datasets (Figure 1b).

Analysis of structural composition showed that both training sets were dominated by X-ray crystal structures. In the v2016 training set, 95.20% of the complexes had crystallographic resolutions of 3.0 Å or better, 2.88% had resolutions poorer than 3.0 Å, and the remaining 1.92% were determined by NMR. The corresponding proportions in the v2020.R1 training set were 95.02%, 3.35%, and 1.62%, respectively. Both CASF-2013 and CASF-2016 consisted entirely of X-ray crystal structures, and all complexes had crystallographic resolutions of 2.5 Å or better. The 1.5–2.0 Å interval was the most frequently represented resolution range in both CASF core sets, accounting for 58.46% and 55.79% of the CASF-2013 and CASF-2016 samples, respectively (Figure 1c). Overall, the training sets provided broader coverage in terms of affinity-label types, structure-determination methods, and crystallographic resolutions. In contrast, the CASF core sets had more homogeneous structural compositions and generally higher crystallographic resolutions.

### 2.2. Analysis of Model Training Process and Convergence Stability

**Figure 2:**
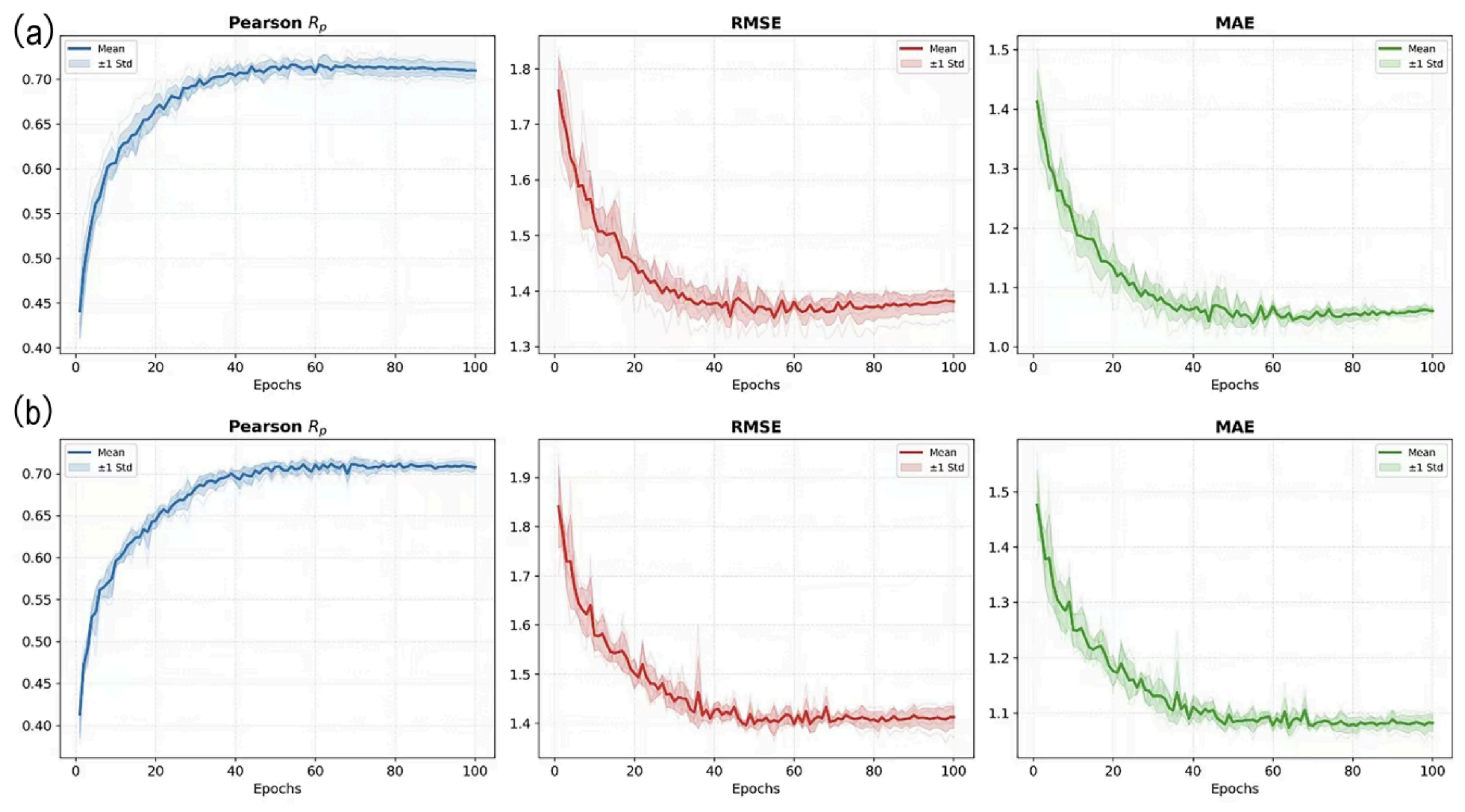
Training process and convergence stability of GAMT-GINE under five-fold cross-validation. (**a**) Performance changes on the internal validation sets when CASF-2013 was used as the external test set. (**b**) Performance changes on the internal validation sets when CASF-2016 was used as the external test set. Solid lines represent the mean results across the five folds, and the shaded regions indicate one standard deviation above and below the mean (±1 SD).

To evaluate the stability and convergence consistency of GAMT-GINE during training, the training data were divided into five mutually exclusive subsets using a fixed random seed, and five-fold cross-validation was subsequently performed. The performance curves obtained on the internal validation set of each fold are shown in Figure 2. The solid lines represent the mean results across the five folds, while the shaded regions indicate one standard deviation above and below the mean (±1 SD). The relatively narrow shaded regions indicate limited variation among the training curves obtained from different data subsets, demonstrating good consistency and convergence stability. As the number of training epochs increased, RMSE and MAE on the internal validation sets generally decreased, whereas *R*_p_ gradually increased. All three metrics approached stable values within 100 epochs. The best-performing model for each fold was selected exclusively according to its performance on the corresponding internal validation set. The external CASF-2013 and CASF-2016 test sets were not used during model training, hyperparameter tuning, or best-model selection. Finally, the best model obtained from each of the five folds was used independently to generate predictions for the CASF test sets. The predictions produced by the five models were then averaged before the final evaluation metrics were calculated. This ensemble strategy reduces predictive variation arising from a single data split or individual model, thereby improving the stability of the external test results.

### 2.3. Predictive Performance on External Test Sets and Cross-Dataset Generalization Evaluation

**Figure 3:**
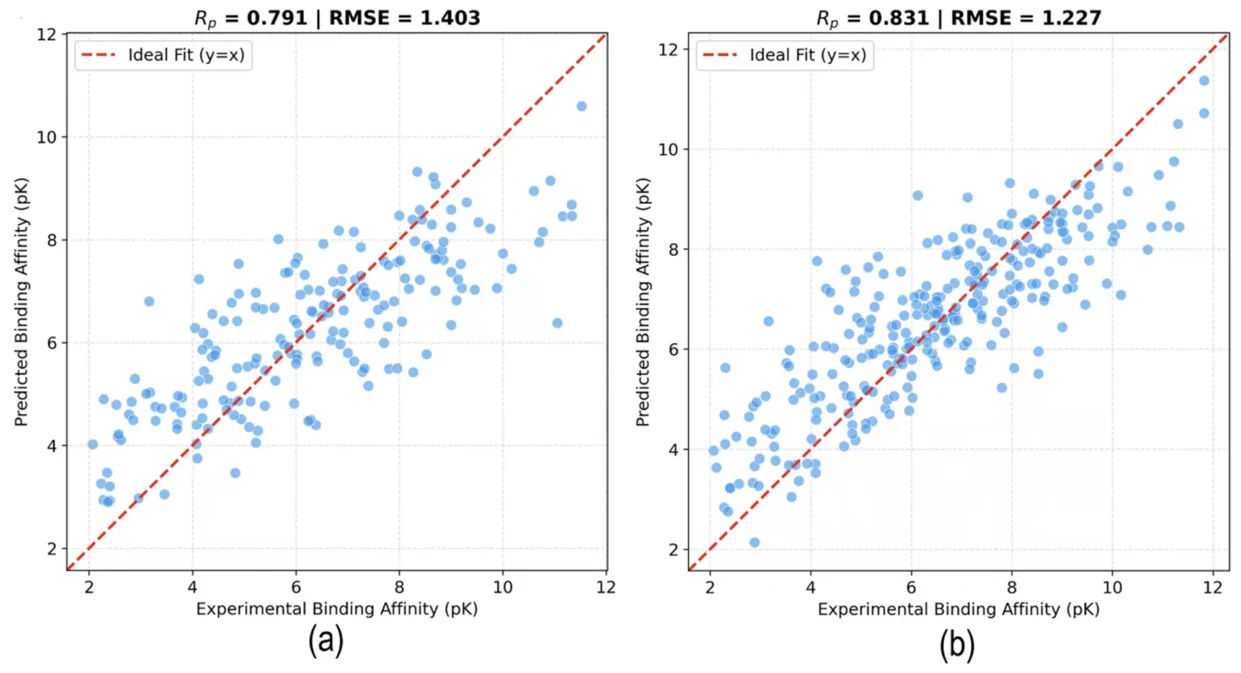
Correlation between experimentally measured and predicted binding affinities of GAMT-GINE on different external test sets. (**a**) CASF-2013 test set. (**b**) CASF-2016 test set. The dashed line represents the identity line.

The predictive performance of GAMT-GINE was further evaluated on two external test sets. As shown in the scatter plots of experimental versus predicted values (Figure 3), most samples are distributed near the identity line, indicating an overall good agreement between the model predictions and the experimentally measured values. On the CASF-2013 test set, GAMT-GINE achieved an *R*_p_ of 0.791 and an RMSE of 1.403, demonstrating good performance in binding affinity prediction (Figure 3a). On the CASF-2016 test set, the model achieved an *R*_p_ of 0.831 and an RMSE of 1.227, likewise showing a strong correlation between the predicted and experimental values and a relatively low prediction error (Figure 3b). GAMT-GINE maintained good predictive performance on both independent benchmark test sets, suggesting a certain degree of cross-dataset generalization capability. It should be noted that CASF-2013 and CASF-2016 may differ in sample composition, protein types, and affinity distributions. Therefore, changes in the evaluation metrics between the two test sets alone cannot be used to determine whether the model performance improved or declined. Accordingly, the predictive accuracy and cross-dataset applicability of GAMT-GINE were evaluated primarily on the basis of its overall performance across both external test sets.

### 2.4. Comparison with State-of-the-Art Models

To further evaluate the binding affinity prediction performance of GAMT-GINE, we compared it with several representative models developed in recent years for protein–ligand binding affinity prediction. The comparative methods included the convolutional neural network model OnionNet [20], the graph neural network models IGN [21], SIGN [30], GIGN [31], and CurvAGN [32], as well as the conventional machine-learning scoring function RF-Score-v3 [33]. These models were compared on the CASF-2013 and CASF-2016 benchmark test sets, and the results are presented in Table 2 and Table 3, respectively.

On the CASF-2013 benchmark test set (Table 2), GAMT-GINE achieved an *R*_p_ of 0.791 and an RMSE of 1.403. Among the models listed, GAMT-GINE obtained the highest *R*_p_ and the lowest RMSE, outperforming OnionNet, SIGN, and IGN overall. Notably, the *R*_p_ values of GAMT-GINE and SIGN were 0.791 and 0.790, respectively, indicating only a marginal difference in correlation performance.

On the CASF-2016 test set (Table 3), GAMT-GINE achieved an *R*_p_ of 0.831 and an RMSE of 1.227, outperforming most of the comparative models listed in the table. Compared with CurvAGN, GAMT-GINE achieved the same *R*_p_, while its RMSE was slightly higher than that of CurvAGN (1.217), indicating that the two models exhibited comparable overall predictive performance on this test set. Compared with IGN and GIGN trained on PDBbind v2020, GAMT-GINE achieved both a higher *R*_p_ and a lower RMSE, demonstrating good predictive accuracy and cross-dataset applicability on the external CASF-2016 test set.

It should be noted that the compared models were not developed under fully consistent conditions with respect to training-data sources, PDBbind versions, sample-filtering criteria, affinity-label composition, and training and validation protocols. Therefore, the results presented in Table 2 and Table 3 are intended primarily as reference comparisons based on previously published metrics. This represents a limitation of the current benchmarking strategy. Future studies should conduct more rigorous model comparisons using unified training data, data partitions, and evaluation protocols.

Overall, the advantage of GAMT-GINE lies in its balance among predictive performance, input representation, utilization of heterogeneous affinity labels, and practical deployment costs. The model employs 17-dimensional basic atomic features and continuous RBF-based distance encoding to learn local spatial relationships within protein–ligand complexes without constructing three-dimensional voxel grids or introducing a large number of high-dimensional hand-crafted descriptors. Its multi-task learning mechanism enables the unified utilization of different affinity-label types, including *K*_d_//*K*_i_ and IC_50_. Computational resource evaluation further showed that a single GAMT-GINE model contains only 237,826 trainable parameters, with a checkpoint size of approximately 0.921 MB; even with a five-model ensemble, the total size of all model files is only 4.605 MB. At a batch size of 32, the five-model ensemble achieved a model-only inference throughput of 360.340 complexes/s. Processing 285 pre-generated graphs and obtaining the final ensemble predictions required an average of 1.996 s, with a peak allocated GPU memory usage of 861.75 MB. Therefore, GAMT-GINE provides a practical option for research scenarios that require a balance among predictive accuracy, ease of input construction, utilization of heterogeneous labels, and convenient deployment on Windows-based workstations.

### 2.5. Ablation Study

**Figure 4:**
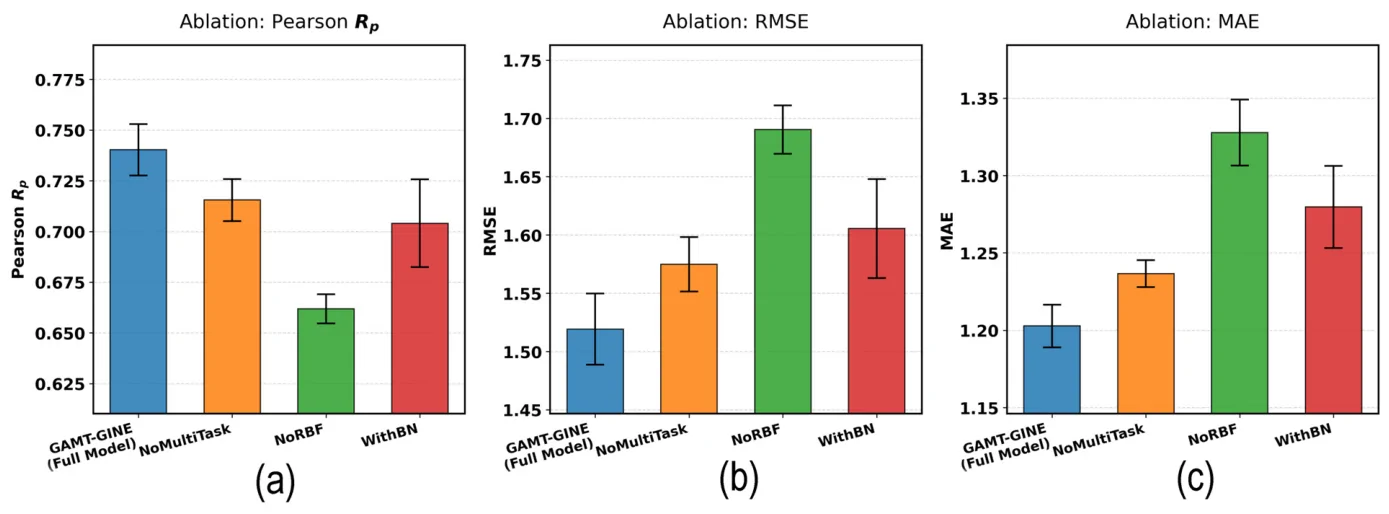
Comparisons of GAMT-GINE and its variants in the ablation study for binding affinity prediction. (**a**) Pearson’s correlation coefficient (*R*_p_); (**b**) root mean square error (RMSE); and (**c**) mean absolute error (MAE).

To evaluate the contributions of the core components of GAMT-GINE to its predictive performance, a series of ablation experiments were conducted (Figure 4). The performance of different model variants was compared under identical experimental conditions by removing or modifying specific components. In addition to the complete model, the following three variants were evaluated:

1. Without MultiTask: The IC_50_ auxiliary task branch was removed, while only the primary task for *K*_d_/*K*_i_ affinity prediction was retained.
2. Without RBF: The radial basis function mapping of interatomic distances was removed from the feature encoding stage.
3. With BN: Conventional batch normalization layers were introduced into the message-passing modules of the graph neural network.

Figure 4 presents the comparative results obtained under five-fold cross-validation, with the error bars representing the standard deviations. The complete model achieved the best performance across all three evaluation metrics. Specifically, it yielded the highest *R*_p_ (Figure 4a) and the lowest RMSE (Figure 4b) and MAE (Figure 4c). A comparison across the three metrics shows that removing the RBF module resulted in the most pronounced performance degradation. This finding indicates that preserving continuous and smoothly varying spatial distance information between atoms is important for accurately capturing the three-dimensional structures of molecular complexes. In addition, the introduction of batch normalization reduced the preservation of intrinsic spatial variations among different molecular complexes, whereas removal of the auxiliary task prevented the model from fully utilizing the large number of samples with alternative affinity labels for auxiliary learning. Both modifications led to reductions in predictive accuracy and stability. Collectively, these results support the rationality and effectiveness of the current GAMT-GINE architecture.

### 2.6. Feature Importance Analysis

To further assess the contributions of atom-type information and three-dimensional distance information to the predictive performance of GAMT-GINE, feature-masking experiments were conducted to examine changes in model performance when either atomic identity information or spatial distance information was removed. Specifically, two masking conditions were considered: node-feature masking, in which the 17-dimensional atom-type features were removed while retaining only the graph topology defined by spatial adjacency between atoms; and edge-feature masking, in which the RBF-encoded three-dimensional distance information was removed while retaining the atom-type features. By comparing the predictive performance of the complete model with that under the two masking conditions, the respective contributions of atom-type information and continuous spatial distance information to model prediction were evaluated.

**Figure 5:**
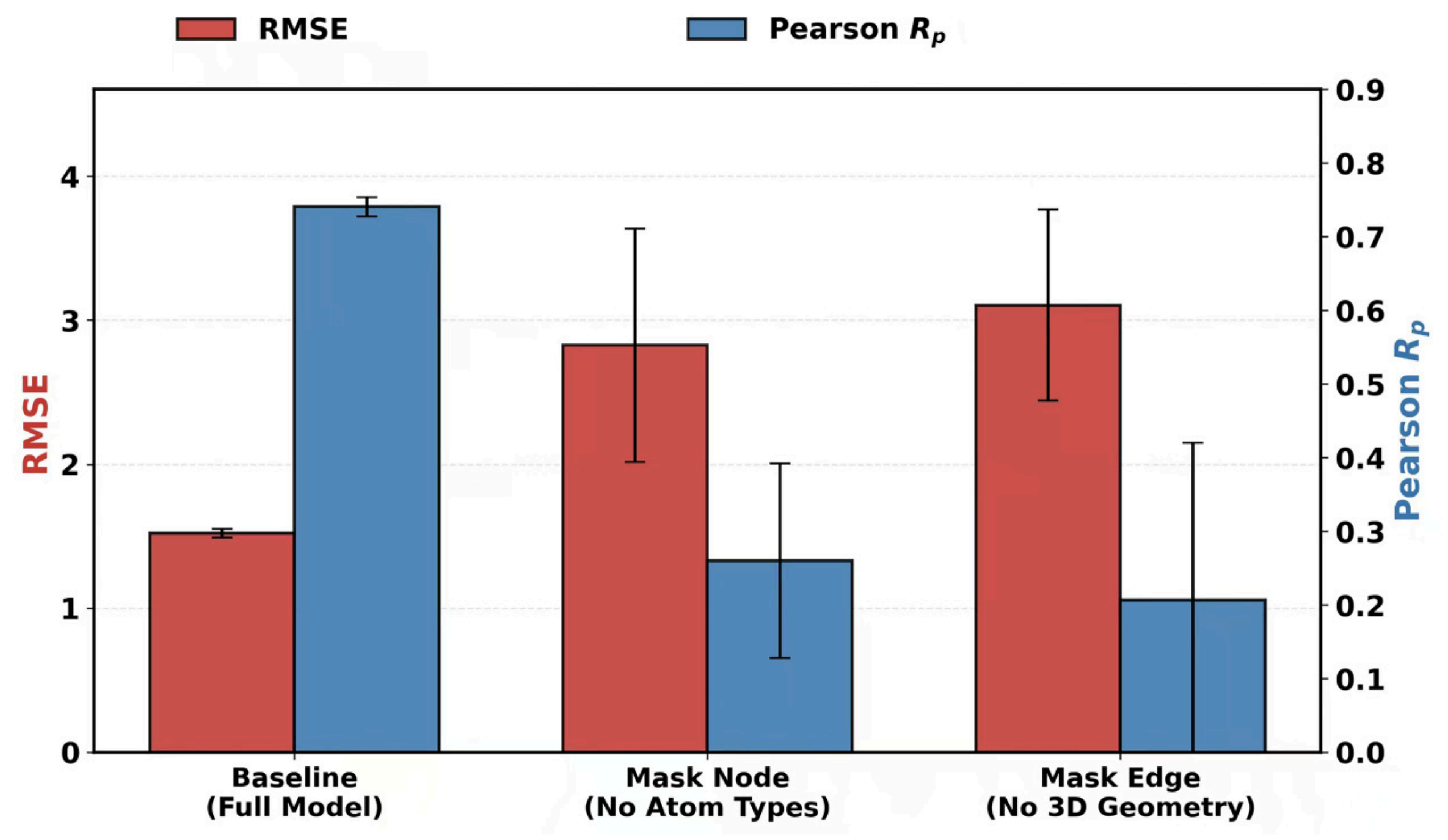
Importance analysis of node and edge features based on a masking strategy.

The five-fold cross-validation results are shown in Figure 5. Removing either component of the minimal feature set resulted in a marked degradation in predictive performance. When the node features were masked, *R*_p_ decreased from the baseline value of 0.740 (±0.013) to 0.260 (±0.132), while RMSE increased to 2.827 (±0.810). These results indicate that explicit atomic chemical identities are essential for distinguishing different types of non-covalent interactions, such as hydrogen bonding and hydrophobic interactions. Masking the edge attributes representing three-dimensional geometric information led to an even greater deterioration in model performance. Specifically, *R*_p_ decreased to 0.207 (±0.214), while RMSE increased to 3.106 (±0.664). This finding indicates that microscopic three-dimensional interatomic distances constitute a key determinant of protein–ligand binding affinity. Knowledge of which atoms participate in binding, without information about their spatial separations, is insufficient for the network to accurately quantify binding strength.

Collectively, these results support the feature design of GAMT-GINE, which integrates basic atomic features with continuous distance encoding. They further demonstrate that the model can achieve good predictive performance without relying on a large number of complex, hand-crafted chemical features.

It should be noted that although the 17-dimensional atomic node vector reduces input complexity, it also constrains the chemical representational scope of the model. This representation distinguishes only 16 predefined elements and a unified “other” category and does not explicitly encode fine-grained properties such as formal charge, bond order, oxidation state, or metal-coordination geometry. Consequently, for nonstandard bioisosteres, subtle differences in electronic effects and bonding patterns may not be adequately represented even when they are composed primarily of common elements. For ligands containing rare elements that are not individually encoded or organometallic centers, different elements may be assigned to the same category. Meanwhile, metal-coordination states cannot be further distinguished, potentially reducing prediction reliability in these specialized chemical systems.

### 2.7. Effects of Data Filtering on Model Performance and Dataset Composition

To distinguish the effects of structural quality filtering and affinity-label filtering on the predictive performance of GAMT-GINE, four training-data processing strategies were evaluated while keeping the model architecture, training parameters, data-splitting procedure, and external test sets unchanged: the original data, structural filtering only, affinity-label filtering only, and combined structural and affinity-label filtering. Structural filtering involved removing structures determined by NMR and non-NMR structures with reported global resolution values greater than 3.0 Å. Affinity-label filtering involved removing samples with affinity qualifiers of “<”, “≤”, or “~”, which represented censored, thresholded, or approximate measurements. CASF-2013 and CASF-2016 were used as fixed independent test sets in all experiments and were not involved in model training, validation, or model selection.

For the CASF-2013 evaluation task, the original training set contained 12,795 complexes. Structural filtering alone removed 246 NMR structures and 368 structures with reported global resolution values greater than 3.0 Å, leaving 12,181 training samples. Affinity-label filtering alone removed 165 samples with uncertain affinity labels, including 86 samples labeled with “<”, 72 with “~”, and 7 with “≤”, leaving 12,630 training samples. After applying both filtering procedures, 12,031 training samples were retained.

For the CASF-2016 evaluation task, the original training set contained 18,752 complexes. Structural filtering alone removed 304 NMR structures and 628 structures with reported global resolution values greater than 3.0 Å, leaving 17,820 training samples. Affinity-label filtering alone removed 270 samples with uncertain affinity labels, including 149 samples labeled with “<”, 114 with “~”, and 7 with “≤”, leaving 18,482 training samples. After applying both filtering procedures, 17,573 training samples were retained.

#### 2.7.1. Effects of Different Filtering Strategies on Predictive Performance

When trained using the original data, GAMT-GINE achieved *R*_p_ = 0.791 and RMSE = 1.403 on CASF-2013 and *R*_p_ = 0.831 and RMSE = 1.227 on CASF-2016.

**Figure 6:**
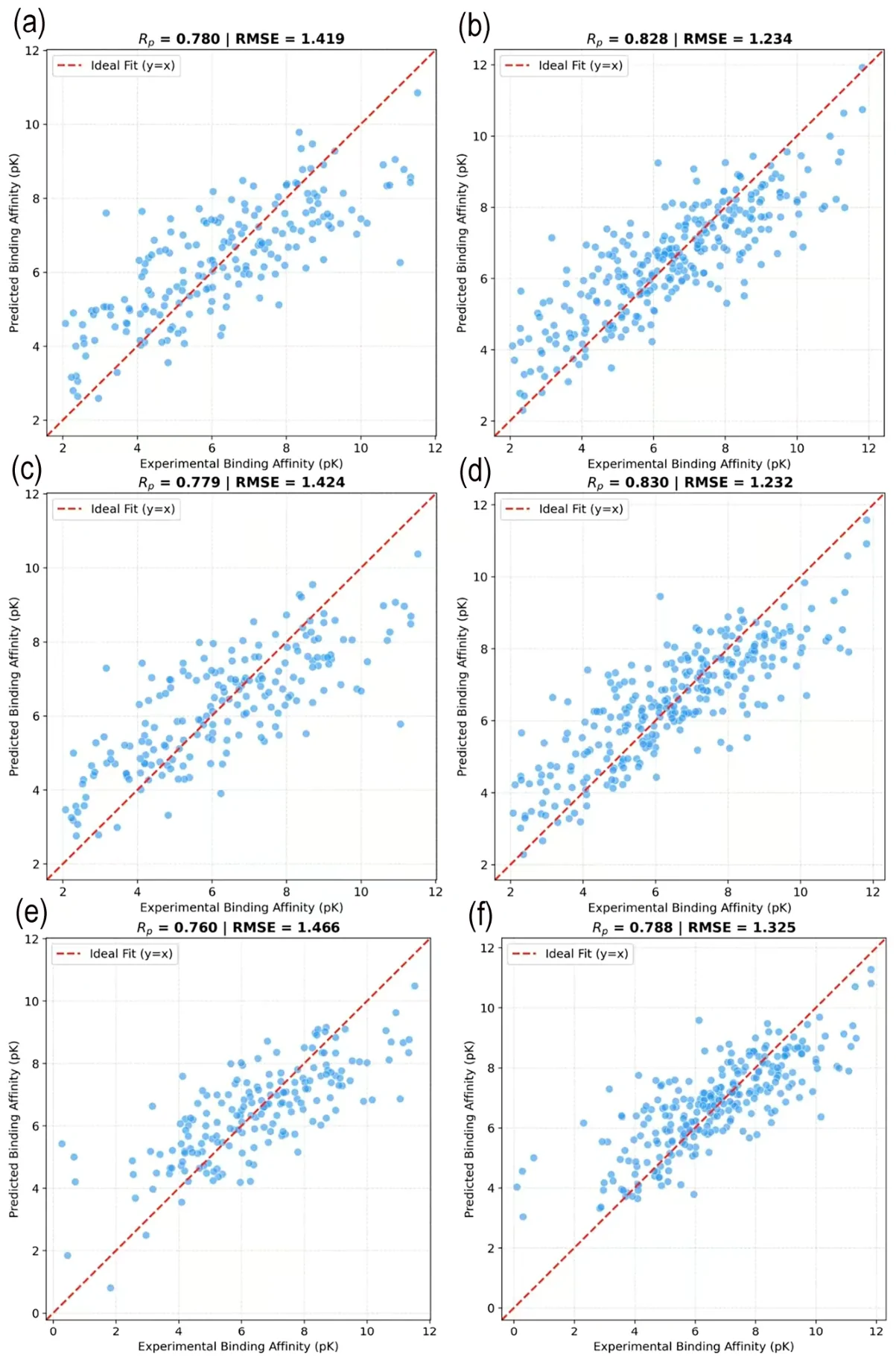
Effects of different data-filtering strategies on the external test performance of GAMT-GINE. (**a**,**b**) Results obtained after structural filtering alone on the CASF-2013 and CASF-2016 test sets, respectively. (**c**,**d**) Results obtained after affinity-label filtering alone on the CASF-2013 and CASF-2016 test sets, respectively. (**e**,**f**) Results obtained after combined structural and affinity-label filtering on the CASF-2013 and CASF-2016 test sets, respectively. Panels (**a**,**c**,**e**) correspond to CASF-2013, whereas panels (**b**,**d**,**f**) correspond to CASF-2016. The dashed line represents the identity line.

After structural filtering alone, the model achieved an *R*_p_ of 0.780 and an RMSE of 1.419 on CASF-2013 (Figure 6a), and an *R*_p_ of 0.828 and an RMSE of 1.234 on CASF-2016 (Figure 6b). Compared with the results obtained using the original data, *R*_p_ decreased by 0.011 and 0.003 on the two test sets, respectively, whereas RMSE increased by 0.016 and 0.007. Although structural filtering removed 4.80% and 4.97% of the corresponding training samples, respectively, the external test performance remained close to that obtained using the original data.

After affinity-label filtering alone, the model achieved an *R*_p_ of 0.779 and an RMSE of 1.424 on CASF-2013 (Figure 6c), and an *R*_p_ of 0.830 and an RMSE of 1.232 on CASF-2016 (Figure 6d). Compared with the results obtained using the original data, *R*_p_ decreased by 0.012 and 0.001 on the two test sets, respectively, whereas RMSE increased by 0.021 and 0.005. Overall, removing uncertain affinity labels alone resulted in only minor changes in predictive performance.

After structural and affinity-label filtering were applied simultaneously, *R*_p_ decreased to 0.760, and RMSE increased to 1.466 on CASF-2013 (Figure 6e). On CASF-2016, *R*_p_ decreased to 0.788, and RMSE increased to 1.325 (Figure 6f). Compared with the results obtained using the original data, *R*_p_ decreased by 0.031 and 0.043 on the two test sets, respectively, whereas RMSE increased by 0.063 and 0.098. The changes caused by combined filtering were more pronounced than those caused by either filtering strategy alone.

The two independent test sets showed a consistent trend. Neither structural filtering nor affinity-label filtering alone improved model performance, although their individual effects were relatively limited. When both filtering procedures were applied simultaneously, the decline in model performance became more pronounced.

#### 2.7.2. Effects of Structural Filtering on Target-Class Composition

Structural filtering removed 932 complexes from the complete set of 19,037 protein–ligand complexes in PDBbind v2020.R1, accounting for 4.90% of the dataset. However, the removal rates differed markedly among target classes. Of the 131 GPCR complexes, 38 were removed, corresponding to a removal rate of 29.0%. Among 147 other membrane-protein complexes, 36 were removed, corresponding to a removal rate of 24.5%. Of the 199 ion-channel complexes, 20 were removed, corresponding to a removal rate of 10.1%. In contrast, the retention rates for proteases and other enzymes were 97.6% and 96.5%, respectively.

The removal of membrane-protein complexes was primarily attributable to the global resolution threshold. Among the removed complexes, 34 GPCR complexes, 34 other membrane-protein complexes, and 18 ion-channel complexes had reported global resolution values greater than 3.0 Å. Compared with the retained non-NMR structures, GPCRs were significantly enriched among structures with global resolution values greater than 3.0 Å, with an odds ratio of 11.09 and a BH-adjusted *p*-value of 1.30 × 10^−20^. Other membrane proteins had an odds ratio of 9.28 and a BH-adjusted *p*-value of 6.45 × 10^−19^, whereas ion channels had an odds ratio of 2.96 and a BH-adjusted *p*-value of 1.78 × 10^−4^.

At the level of unique UniProt targets, structural filtering completely removed 6 of the 48 GPCR targets, 8 of the 36 ion-channel targets, and 17 of the 62 other membrane-protein targets from the retained dataset. The corresponding unique-target retention rates were 87.5%, 77.8%, and 72.6%, respectively. The unique-target retention rates for kinases, proteases, and other enzymes were 95.8%, 97.1%, and 95.8%, respectively.

Exclusion of NMR structures showed a different selection pattern. Small soluble proteins were defined as proteins without evidence of membrane association and with PDB construct lengths of no more than 200 residues. Among the 304 excluded NMR complexes, 278 were classified as small soluble proteins, accounting for 91.4% of the group. In contrast, 4121 of the 18,105 retained structures belonged to this category, accounting for 22.8%. Small soluble proteins were significantly enriched in the excluded NMR group, with an odds ratio of 37.53 and a BH-adjusted *p*-value of 2.05 × 10^−14^. The median PDB construct length was 105 residues in the NMR group and 267 residues in the retained group, with a Cliff’s δ of −0.840. Analyses using thresholds of 150 and 250 residues produced results in the same direction.

These findings indicate that, although structural filtering removed only approximately 5% of the complexes, its effects were not uniformly distributed. The global resolution threshold had relatively large effects on GPCRs, other membrane proteins, and ion channels, whereas exclusion of NMR structures primarily reduced the representation of small soluble proteins and shorter protein constructs.

#### 2.7.3. Effects of Affinity-Label Filtering on Affinity Distributions

To determine whether the removal of uncertain affinity labels altered the affinity composition of the training data, the *pK* distributions of samples with exact and uncertain labels were further compared.

In the training data corresponding to the CASF-2013 evaluation, samples with exact labels had a median *pK* of 6.46, whereas those with uncertain labels had a median *pK* of 7.30. Among samples with exact labels, 20.0% were located in the *pK* ≥ 8 region, compared with 45.5% of samples with uncertain labels. In the training data corresponding to the CASF-2016 evaluation, samples with exact labels had a median *pK* of 6.44, whereas those with uncertain labels had a median *pK* of 7.60. The proportions of samples in the *pK* ≥ 8 region were 19.6% and 47.8% for the exact- and uncertain-label groups, respectively.

After removing samples with uncertain labels, the overall *pK* range of the remaining exact-label samples was still 0.40–15.22. Therefore, affinity-label filtering did not alter the minimum or maximum values of the training data but reduced the sample density in the higher-*pK* region and in regions represented by censored, thresholded, or approximate measurements.

Model performance changed only slightly after affinity-label filtering alone, suggesting that this distributional change by itself was insufficient to cause a marked decline in external test performance. Under combined filtering, however, changes in the affinity distribution occurred together with reductions in structural and target coverage, which may jointly decrease the amount of structure–affinity information contained in the training data.

#### 2.7.4. Summary

Structural filtering or affinity-label filtering alone caused only minor changes in predictive performance, whereas their combined application resulted in more pronounced performance declines on both CASF-2013 and CASF-2016. These results indicate that the effects of data filtering on model performance cannot be attributed to a single factor.

Affinity-label filtering reduced the density of samples in the higher-*pK* region and in regions containing uncertain measurements, whereas structural filtering reduced the representation of certain membrane proteins, small soluble proteins, and unique targets. When both filtering procedures were applied simultaneously, the number of training samples, affinity distributions, and structural composition changed concurrently, potentially further reducing the coverage of the external test sets by the training data.

### 2.8. Effect of Training–Test Data Similarity on Predictive Performance

**Figure 7:**
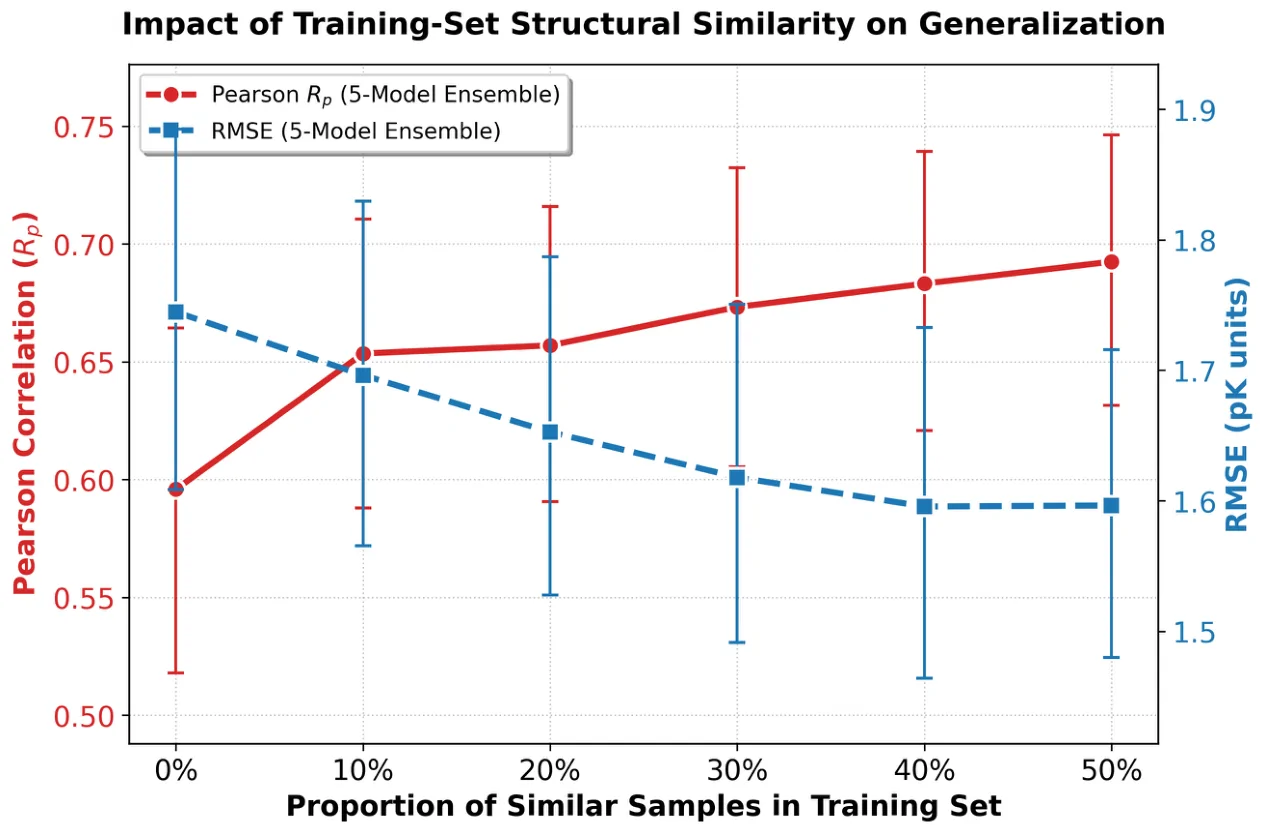
Effects of similar sample ratio in the training set on model prediction performance. Error bars represent 95% confidence intervals obtained by bootstrap resampling of the 285 test complexes.

To investigate the effect of protein three-dimensional structural similarity between the training and test sets on model predictive performance, six equally sized training subsets were constructed, each containing 2600 protein–ligand complexes. The proportions of samples structurally similar to the CASF-2016 test proteins were set to 0%, 10%, 20%, 30%, 40%, and 50%, respectively. Protein structural similarity was quantified using TM-score, and samples with a TM-score greater than 0.5 were defined as structurally similar samples [34]. For each training subset, the model was trained using five different training–validation splits and evaluated on the independent CASF-2016 test set. During external testing, the predictions generated by the five models for the same complex were first averaged, after which *R*_p_ and RMSE were calculated based on the ensemble predictions. The results are shown in Figure 7.

As the proportion of structurally similar samples in the training set increased, the predictive performance of GAMT-GINE generally showed a continuous improvement trend. When the proportions of structurally similar samples were 0%, 10%, 20%, 30%, 40%, and 50%, the corresponding *R*_p_ values were 0.596, 0.654, 0.657, 0.673, 0.683, and 0.693, respectively, whereas the corresponding RMSE values were 1.744, 1.696, 1.653, 1.618, 1.596, and 1.596. When the proportion of structurally similar samples increased from 0% to 50%, *R*_p_ increased by 0.097, and RMSE decreased by 0.148, indicating that increasing the number of training samples structurally similar to the test proteins helps improve the correlation between predicted and experimental values and reduce the overall prediction error.

### 2.9. Relationship with Emerging Biomolecular Structure Prediction Methods

In recent years, methods such as AlphaFold 3 [35] and RoseTTAFold All-Atom [36] have extended biomolecular structure prediction to complex systems containing proteins, small-molecule ligands, nucleic acids, ions, and other molecular components. These methods typically take protein sequences and associated molecular components as input to generate candidate all-atom three-dimensional complex structures together with corresponding structural confidence information. In contrast, GAMT-GINE takes a given protein–ligand complex conformation as input and predicts *pK*_d_/*pK*_i_ values based on atom types and protein–ligand spatial contact relationships within the complex.

These approaches correspond to two complementary computational tasks: complex structure generation and binding affinity estimation. The outputs of AlphaFold 3 and RoseTTAFold All-Atom mainly include atomic coordinates, candidate conformations, and structural confidence information, and their performance is evaluated using metrics such as ligand RMSD, LDDT, DockQ, and other measures of structural or ligand-pose accuracy, depending on the specific task. GAMT-GINE, by contrast, outputs continuous binding affinity predictions, and its performance is assessed using *R*_p_, RMSE, and MAE to quantify agreement between predicted and experimentally measured values. A comparison of the three methods in terms of task objectives, input information, model outputs, evaluation metrics, and potential roles in practical workflows is provided in Table 4.

In practical applications, complex structure prediction and binding affinity estimation can form a sequential computational workflow. For targets lacking experimentally determined structures, AlphaFold 3 or RoseTTAFold All-Atom may first be used to generate candidate protein–ligand complex conformations, after which GAMT-GINE can be applied to estimate the binding affinities of the corresponding protein–ligand systems. Because GAMT-GINE was trained primarily on experimentally resolved complex structures from PDBbind, conformations generated by structure prediction methods may differ from experimental structures in ligand pose, local geometry, or overall conformational state, potentially resulting in a shift in the input data distribution. Therefore, the applicability and predictive performance of GAMT-GINE when using predicted structures as input still require further evaluation on independent test sets or through prospective validation.

### 2.10. Retrospective Multi-Target Candidate Prioritization and a Representative PDE10A Case

**Figure 8:**
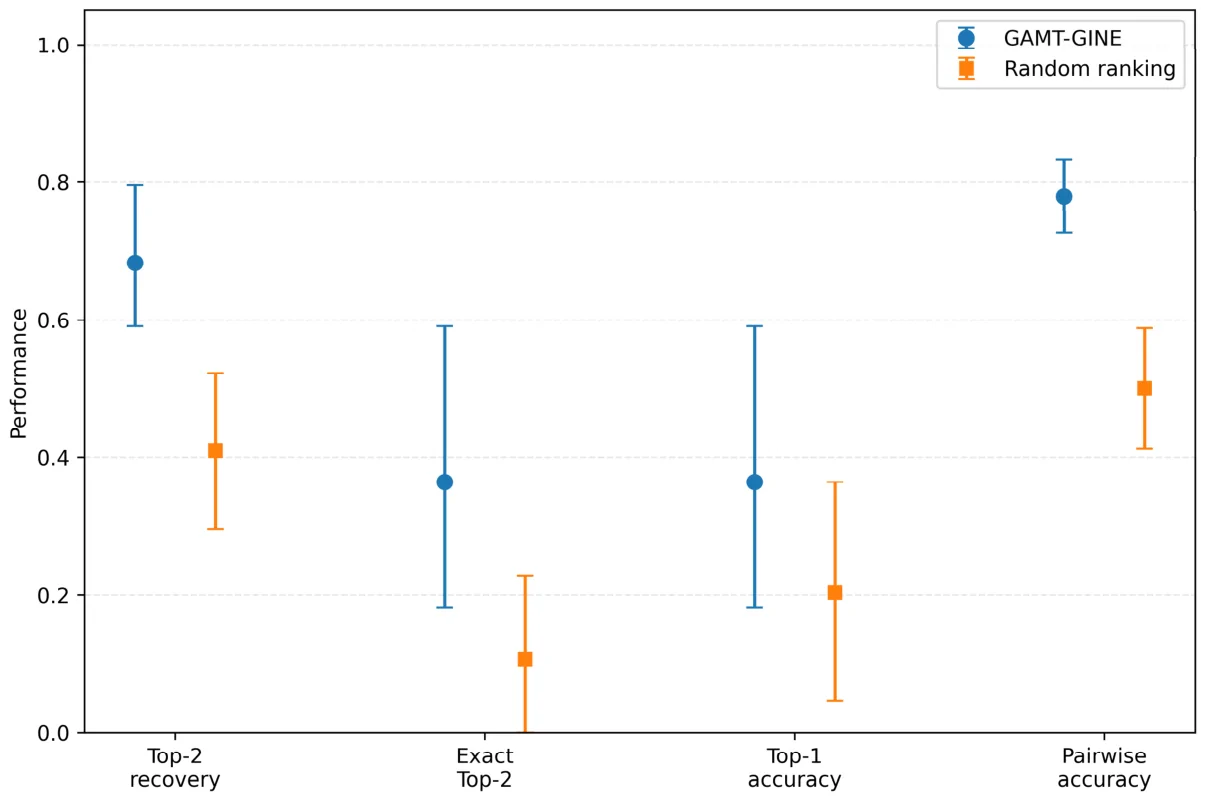
Performance of GAMT-GINE in retrospective multi-target candidate prioritization. Error bars represent the bootstrap 95% confidence intervals for GAMT-GINE and the 95% simulation intervals for random ranking, respectively.

To further evaluate the potential utility of GAMT-GINE for candidate prioritization, a retrospective multi-target analysis was performed using the CASF-2016 test set. The 285 protein–ligand complexes were grouped according to target UniProt identifiers, resulting in 67 target groups. Among them, 53 groups met the screening criteria of complete structures, unambiguous target annotation, at least four complexes per group, and an experimental affinity range of no less than 1.5 *pK* units. After further requiring a consistent affinity measurement type within each target group, 22 groups containing only *K*_d_ or *K*_i_ measurements were retained, comprising a total of 108 protein–ligand complexes. Within each target, candidates were ranked in descending order according to the *pK* values predicted by GAMT-GINE. The macro-averaged Top-2 retention rate was 0.682, with a 95% confidence interval of 0.591–0.795, exceeding the random-ranking baseline of 0.409 (one-sided empirical *p* < 0.001). Among the 22 targets, both experimentally determined Top-2 candidates were included in the predicted Top-2 for 8 targets, while one of the two was retained for each of the remaining 14 targets. The Exact Top-2 recovery rate was 36.4%, higher than the 10.6% obtained under random ranking (*p* = 0.0018). The macro-averaged pairwise ranking accuracy was 0.779, with a 95% confidence interval of 0.726–0.832, exceeding the random-ranking baseline of 0.500 (one-sided empirical *p* < 0.001). The Top-1 accuracy was 36.4%, compared with 20.3% under random ranking, although the difference did not reach statistical significance (*p* = 0.0579). When only the top two predicted candidates were retained for each target, the candidate list was reduced by an average of 59.1%. The corresponding results are shown in Figure 8.

PDE10A, with UniProt accession Q9Y233, was selected as the representative case according to the predefined selection rule and comprised five protein–ligand complexes. The two complexes with the highest experimental affinities, 5C2H and 3UI7, were both retained among the top two model-predicted candidates, reducing the candidate list from five to two. Among the 10 pairwise comparisons formed by the five candidates, the model correctly reproduced the relative affinity ordering for 9 pairs. Only the ordering between 5C2H and 3UI7 was reversed, whereas the predicted rankings of the remaining three complexes, 3UUO, 5C28, and 4LLX, were consistent with their experimental rankings. Further comparison of the experimental and predicted values showed that the model largely preserved the overall affinity ranking of the PDE10A candidates, although the predicted affinity range was compressed. For 5C2H, the experimental *pK* was 11.086, and the predicted value was 8.074; for 3UI7, the corresponding values were 9.000 and 8.121; for 3UUO, 7.959 and 7.586; for 5C28, 5.658 and 5.039; and for 4LLX, 2.886 and 4.238. Overall, the experimental *pK* values ranged from 2.886 to 11.086, whereas the predicted values ranged from 4.238 to 8.121. The experimental and predicted affinities of the PDE10A complexes are summarized in Table 5.

Overall, GAMT-GINE showed favorable performance in retaining high-affinity candidates and preserving within-target relative rankings across multiple targets. However, further improvement remains necessary for identifying the single highest-affinity candidate and for quantitatively predicting extreme affinity values.

## 3. Materials and Methods

### 3.1. Dataset Preparation

The PDBbind database was used as the source of protein–ligand complex structures and experimentally measured binding-affinity data. For each complex, PDBbind provides the corresponding three-dimensional protein and ligand structure files, together with experimental measurements such as the dissociation constant (*K*_d_), inhibition constant (*K*_i_), or half-maximal inhibitory concentration (IC_50_).

The primary training data were obtained from the officially released PDBbind v2020.R1 dataset. This dataset contains 19,037 protein–ligand complexes that were included in PDBbind v2020 and remained available in PDBbind v2024. In addition, it contains 143 nucleic-acid complexes, 2798 protein–protein complexes, and 1032 protein–nucleic-acid complexes; only the protein–ligand complexes were used in this study. The structure files provided in PDBbind v2020.R1 were reprocessed using the PDBbind v2024 data-processing workflow, and the structural and binding information of some complexes was updated in the index files. Compared with the direct use of the original PDBbind v2020 files, this dataset provides protein and ligand structure files processed in a more consistent manner across different complexes.

To enable comparisons with previous studies on protein–ligand binding-affinity prediction, two evaluation datasets based on CASF-2013 and CASF-2016 were constructed. For the CASF-2013 evaluation task, the protein–ligand complexes in PDBbind v2016 were first used as the candidate set and matched to PDBbind v2020.R1 according to their PDB identifiers. The intersection contained 12,990 candidate complexes present in both PDBbind v2016 and PDBbind v2020.R1. The 195 complexes in the CASF-2013 core set were subsequently excluded according to their PDB identifiers, yielding 12,795 training complexes, while the 195 CASF-2013 core-set complexes were retained as a fixed independent test set. For the CASF-2016 evaluation task, the 19,037 protein–ligand complexes in PDBbind v2020.R1 were used as the initial candidate set. After excluding the 285 complexes in the CASF-2016 core set according to their PDB identifiers, 18,752 training complexes were retained, while the 285 CASF-2016 core-set complexes were used as a fixed independent test set. Verification based on PDB identifiers confirmed that there was no complex-level overlap between the training set and the corresponding CASF test set in either evaluation task.

Regarding data filtering, the original dataset was defined as protein–ligand complexes with valid PDB identifiers for which *K*_d_, *K*_i_, or IC_50_ records could be successfully parsed from the corresponding PDBbind index files. During construction of the original dataset, no additional filtering was performed according to the experimental structure-determination method, structural resolution, or affinity qualifier. Therefore, the original dataset retained structures covering different crystallographic resolution ranges, NMR-derived structures, and affinity records with either exact or non-exact qualifiers. Additional filtering based on structural quality and label precision was applied only in the subsequent data-filtering comparison experiments. The specific filtering criteria, numbers of excluded samples, and sizes of the resulting datasets are described separately in the section entitled “Effects of Data Filtering on Model Performance and Dataset Composition.”

All affinity values were first converted to molar concentrations and then transformed to the negative logarithmic scale as follows:

(1) $$\mathrm{pK}=-\log_{10}(C_{\mathrm{aff}})$$

where $C_{\mathrm{aff}}$ denotes the *K*_d_, *K*_i_, or IC_50_ value expressed in $\mathrm{mol}\cdot L^{-1}$. Because a negative base-10 logarithmic transformation was applied, a tenfold decrease in the original molar value corresponds to an increase of 1 *pK* unit. For *K*_d_, a higher *pK* value corresponds to a lower dissociation constant and generally indicates stronger protein–ligand binding. For *K*_i_ and IC_50_, higher *pK* values correspond to lower inhibition constants and half-maximal inhibitory concentrations, respectively, indicating stronger inhibitory effects under the corresponding experimental conditions. This logarithmic scale transforms experimental measurements spanning multiple orders of magnitude into a numerical range more suitable for statistical analysis and model learning. The *K*_d_ and *K*_i_ values were combined as training labels for the primary binding-affinity prediction task, whereas IC_50_ values were used as labels for the auxiliary task to support subsequent multi-task model training. To maintain consistency with the graph-construction and model-training workflows, the affinity type and *pK* label of each retained complex were obtained from the uniformly generated PDBbind v2020.R1 label table.

### 3.2. Model Validation and Independent Testing Strategy

This study adopted a two-stage evaluation strategy consisting of five-fold cross-validation within the training set and independent testing on the CASF core sets. Five-fold cross-validation was performed exclusively within the training sets defined in Section 3.1. The CASF-2013 and CASF-2016 core sets were used as fixed independent test sets and were strictly isolated from model training, internal validation, and model selection.

At the complex level, the training complexes were divided into five mutually exclusive subsets using KFold, with n_splits = 5, shuffle = True, and random_state = 42. The setting random_state = 42 was used to fix the sample-shuffling and fold-assignment results, thereby ensuring consistent data partitions when the input sample set remained unchanged. In each cross-validation iteration, four subsets were used for model-parameter learning, whereas the remaining subset was used as the internal validation set. Each training complex served as an internal validation sample in only one fold, ensuring that the same complex did not simultaneously participate in training and internal validation within any cross-validation iteration.

During training, the internal validation set was used to evaluate model performance at each epoch, adjust the learning rate, and select the optimal model checkpoint. At the end of each training epoch, the model generated affinity predictions for the corresponding internal validation set, and the *R*_p_, RMSE, and MAE were calculated. The learning-rate scheduler monitored the validation *R*_p_. For each fold, the model parameters corresponding to the highest validation *R*_p_ were retained, ultimately yielding five optimal models based on different training–validation partitions.

The CASF-2013 and CASF-2016 core sets were not involved in the five-fold partitioning, model training, internal validation, learning-rate adjustment, or optimal-model selection. They were used only for final independent evaluation after all five folds had been trained. During independent testing, each of the five optimal fold models generated a prediction for every CASF test complex, and the arithmetic mean of the five predictions was used as the final ensemble prediction for that complex:

(2) $${\hat{y}}_{i}^{\mathrm{ens}}=\frac{1}{5}\sum_{f=1}^{5}{\hat{y}}_{i,f}$$

where ${\hat{y}}_{i,f}$ denotes the prediction generated by the f-th fold model for the i-th test complex, and ${\hat{y}}_{i}^{\mathrm{ens}}$ denotes the corresponding five-model mean ensemble prediction.

### 3.3. Model Evaluation Metrics

To comprehensively and objectively quantify the model’s performance in the binding affinity prediction task and adhering to the evaluation specifications of mainstream benchmarks in this field, this study employed three core statistical metrics to evaluate the prediction results: the Pearson correlation coefficient (*R*_p_), root mean square error (RMSE), and mean absolute error (MAE).

*R*_p_ is used to measure the degree of linear correlation between the predicted affinities and the true experimental values. The RMSE and MAE quantify the absolute error level of the model’s predictions from different dimensions. These metrics were calculated as follows:

(3) $$R_{p}=\frac{\sum_{i=1}^{N}(f(x_{i})-\bar{f(x)})(Y_{i}-\bar{Y})}{\sqrt{\sum_{i=1}^{N}(f(x_{i})-\bar{f(x)}{)}^{2}}\sqrt{\sum_{i=1}^{N}(Y_{i}-\bar{Y}{)}^{2}}}$$

(4) $$RMSE=\sqrt{\frac{1}{N}\sum_{i=1}^{N}(}Y_{i}-f(x_{i}){)}^{2}$$

(5) $$MAE=\frac{1}{N}\sum_{i=1}^{N}\left|Y_{i}-f\left(x_{i}\right)\right|$$

In the above formulas, N denotes the total number of samples in the test set, and i denotes the sample index, where i = 1, 2, …, N. $x_{i}$ represents the input features of the i-th protein–ligand complex, and $f(x_{i})$ denotes the affinity value predicted by the model for the i-th sample. $\bar{f(x)}$ represents the mean of all predicted values. $Y_{i}$ denotes the experimentally measured affinity value of the i-th sample, and $\bar{Y}$ represents the mean of all experimentally measured values.

### 3.4. Construction of the Spatial Atomic Graph and Feature Engineering

To enable graph neural network processing of the three-dimensional structures of protein–ligand complexes, each complex was converted into an atom-level graph composed of nodes and edges. Specifically, each protein or ligand atom was represented as a node, and edges were established between spatially proximal protein and ligand atoms. Accordingly, the graph mainly represents two types of information: node features describe the atom types involved in local spatial contacts, whereas edge features describe the spatial proximity and three-dimensional distances between protein and ligand atoms. An atom-level bipartite graph representation was adopted, in which nodes were divided into protein atoms and ligand atoms, and only intermolecular connections between the two node sets were included. Intramolecular covalent bonds within the protein or ligand were not represented as graph edges.

Node feature extraction and atom-type encoding. To reduce dependence on complex hand-crafted chemical descriptors, basic elemental categories were used as atomic node features. RDKit was used to parse ligand structures and atomic coordinates, while a customized spatial parsing procedure was used to extract three-dimensional coordinates and elemental information for protein atoms from ATOM records in the protein PDB files. The model predefined 16 element types—C, N, O, F, P, S, Cl, Br, I, B, Si, Fe, Zn, Mg, Ca, and Mn—and all elements not included in these categories were assigned to an “other” class, resulting in a 17-dimensional one-hot node feature vector. In this encoding scheme, each dimension corresponds to a predefined element category, allowing the model to distinguish among different atomic element types. Notably, this node representation explicitly encodes only elemental identity and does not include finer-grained chemical properties such as formal charge, bond order, aromaticity, hybridization state, oxidation state, or metal coordination environment.

Topological edge construction and spatial distance features. To focus the graph representation on local contacts between the protein and ligand, Euclidean distances were calculated for all protein–ligand atom pairs, and 5.0 Å was used as the intermolecular edge cutoff. A bidirectional edge was established between protein atom i and ligand atom j when their spatial distance satisfied d_ij_ < 5.0 Å; atom pairs not meeting this criterion were not connected. This cutoff was used to define the local spatial environment around the binding interface, allowing the message-passing process to focus on spatial proximity patterns potentially associated with local non-covalent protein–ligand interactions while reducing the computational cost introduced by irrelevant long-range atom pairs. For each established edge, the actual interatomic distance was retained as a one-dimensional continuous edge feature and subsequently encoded using radial basis functions in the GAMT-GINE model to provide a finer representation of subtle spatial distance variations between atom pairs. The detailed composition of node and edge features is summarized in Table 6.

### 3.5. GAMT-GINE Model Architecture

**Figure 9:**
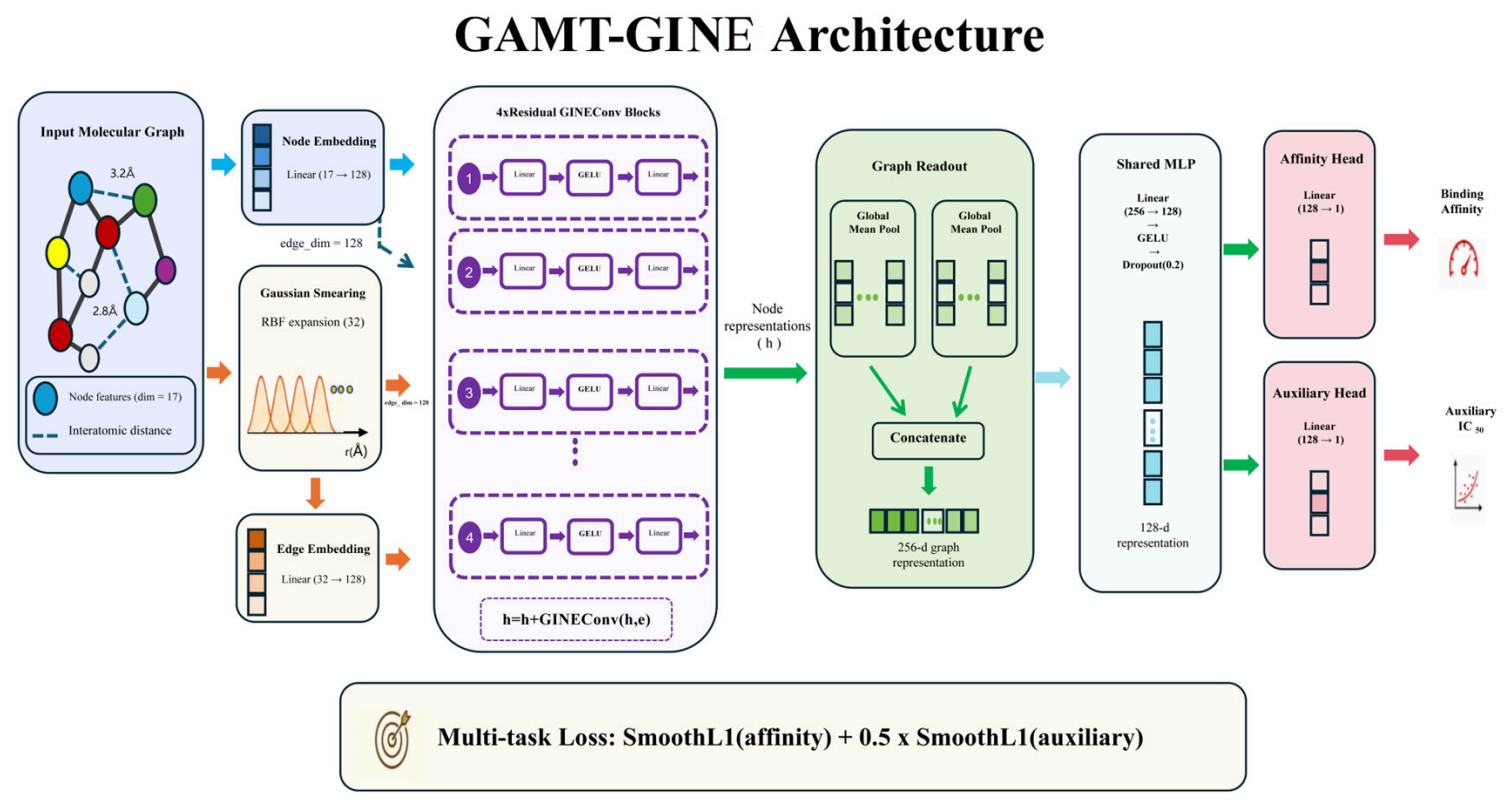
Architecture of the GAMT-GINE model.

Overall, GAMT-GINE consists of three main stages: continuous spatial feature encoding, graph message passing, and global feature aggregation. First, continuous spatial distances between protein and ligand atoms are transformed into high-dimensional spatial features suitable for neural network learning. Next, GINE convolutions are used to propagate and integrate node and edge information among connected atomic nodes. Finally, the local representations of all atomic nodes are aggregated into a fixed-length feature vector describing the entire protein–ligand complex, which is then used for downstream binding affinity prediction. The overall architecture of the model is shown in Figure 9.

During continuous spatial feature encoding, Gaussian radial basis functions are applied to expand the one-dimensional interatomic distances retained in Section 3.4. Thirty-two Gaussian centers are evenly distributed over the range of 0–5.0 Å, converting each scalar edge distance into a 32-dimensional continuous feature representation. This encoding allows similar distances to produce similar responses in feature space and enables subtle distance variations to be reflected smoothly in the subsequent network input, thereby providing finer-grained geometric information for learning local protein–ligand spatial contact patterns.

During graph message passing, the model employs four GINE convolutional layers to progressively update atomic node representations. GINE convolution incorporates edge features during node feature aggregation, allowing each atomic node to integrate both the atom-type information of its connected neighbors and the corresponding spatial distance information. As the network depth increases, information from progressively larger local neighborhoods is integrated to capture protein–ligand interaction features. Within each GINE layer, a multilayer perceptron with GELU activation is used for nonlinear feature transformation. Unlike conventional graph neural network architectures, batch normalization is not introduced into the message-passing module in order to reduce the potential influence of batch-level feature standardization on the scale of spatially related features across different complexes.

During global feature aggregation, protein–ligand complexes containing different numbers of atoms must be transformed from variable-sized node representations into fixed-length complex-level representations. To achieve this, global mean pooling and global max pooling are applied to all node features, and their outputs are concatenated. Mean pooling summarizes the average response of node features across the entire complex, whereas max pooling retains highly responsive local node features. The resulting combined representation forms a complex-level feature vector, which is subsequently passed through a shared fully connected layer with Dropout regularization and serves as the shared complex-level representation for the downstream primary and auxiliary prediction branches.

### 3.6. Multi-Task Loss Function

Experimental affinity measurements are not uniform across protein–ligand complexes in PDBbind. Some samples are labeled with *K*_d_ or *K*_i_, whereas others provide only IC_50_ values. If the model were trained exclusively on *K*_d_ and *K*_i_ samples, the protein–ligand structural information contained in IC_50_-labeled samples would not contribute to model learning. To make fuller use of these heterogeneous experimental data, a multi-task learning strategy was adopted in which the primary affinity prediction task and the auxiliary task share the same graph-based feature-extraction network.

Specifically, two independent output branches were introduced after the shared GINE feature-extraction module and a fully connected representation layer. The primary task predicts *pK* values corresponding to *K*_d_ and *K*_i_, whereas the auxiliary task predicts *pK* values corresponding to IC_50_. The two tasks share the aforementioned complex-level structural representation but generate predictions and compute losses through separate output branches. In this way, the model can exploit the protein–ligand structural information common to different types of experimental measurements without assuming that IC_50_ and *K*_d_/*K*_i_ have identical physical meanings.

The overall optimization objective of the model is constructed based on the Smooth L1 loss, and its joint multi-task loss function is defined as follows:

(6) $$L_{\mathrm{total}}=L_{\mathrm{SmoothL}1}(Y_{\mathrm{aff}},{\hat{Y}}_{\mathrm{aff}})+\lambda \cdot L_{\mathrm{SmoothL}1}(Y_{\mathrm{aux}},{\hat{Y}}_{\mathrm{aux}})$$

where $Y_{aff}$ and $Y_{aux}$ represent the true labels for the main and auxiliary tasks, respectively; $\hat{Y}$ denotes the model predictions, and the weight coefficient λ is set to 0.5. This multi-task learning mechanism fully leverages the large-scale secondary label data without significantly increasing the computational overhead of the model. This is equivalent to introducing additional supervision signals to the network, which imposes an effective data-driven regularization constraint on the feature extraction of the backbone network, further enhancing the generalization robustness of the model on the core prediction task.

### 3.7. Training Protocol and Hyperparameter Settings

The training protocol and hyperparameter settings were standardized. GAMT-GINE employed 17-dimensional atomic node features and four graph neural network layers for message passing, with the hidden feature dimension of each layer set to 128. To encode continuous spatial relationships within the binding pocket, interatomic distances within a cutoff radius of 5.0 Å were expanded using 32 Gaussian radial basis functions. The Gaussian error linear unit (GELU) was used as the activation function for nonlinear feature transformations.

Model training followed the five-fold cross-validation strategy described in Section 3.2. The model in each fold was trained for a maximum of 100 epochs with a batch size of 16. AdamW was used as the optimizer, with an initial learning rate of 1 × 10^−4^ and a weight decay of 1 × 10^−2^. To reduce the risk of overfitting, Dropout with a rate of 0.2 was applied to the shared fully connected layer preceding the primary and auxiliary prediction heads.

During training, a ReduceLROnPlateau learning-rate scheduler was employed, with the internal validation *R*_p_ used as the monitored metric. When the validation *R*_p_ did not improve for 10 consecutive epochs, the current learning rate was multiplied by 0.5. Smooth L1 loss was used for both the primary and auxiliary tasks to reduce the influence of large prediction errors on model optimization. The total loss was defined as a weighted combination of the primary-task and auxiliary-task losses, with the auxiliary-task loss weight set to 0.5. For each fold, the model checkpoint corresponding to the highest validation *R*_p_ was retained. During final inference, an ensemble-learning strategy was adopted, and the predictions generated by the best-performing models retained from the five folds were averaged to obtain the final output. The principal hyperparameters and training settings are summarized in Table 7.

### 3.8. Computational Resources and Runtime Efficiency

To evaluate the computational requirements of GAMT-GINE in practical applications, computational resource usage and runtime efficiency were assessed under Microsoft Windows Server 2022 Datacenter. The test platform provided access to 8 CPU cores and 16 logical processors of an AMD EPYC 7542 processor, together with 32 GB of system memory and an NVIDIA GeForce RTX 4090 GPU with 24 GB of memory. The benchmark consisted of 285 protein–ligand complexes from the CASF-2016 test set, and the final predictions were obtained using an ensemble of the five cross-validation models. All models were set to evaluation mode, and GPU warm-up was completed before formal timing. The principal results are summarized in Table 8.

A single GAMT-GINE model contained 237,826 trainable parameters with a checkpoint file size of approximately 0.921 MB. The total size of the weight files for the five-model ensemble was 4.605 MB. Using eight CPU worker processes, the mean total time required to construct graphs for all 285 complexes from the original protein PDB and ligand SDF files was 14.049 ± 0.834 s. All 285 test complexes were successfully processed in each of the three complete runs.

At a batch size of 1, the five-model ensemble achieved a mean model-only inference latency of 14.660 ± 2.152 ms per complex. At a batch size of 32, the mean model-only inference throughput was 360.340 complexes/s. When graph-file loading, batch construction, CPU-to-GPU data transfer, and the forward passes of all five ensemble models were included in the timing, prediction of all 285 complexes required an average of 1.996 ± 0.157 s. The peak CPU resident set size (RSS) during inference was 881.54 MB, and the peak allocated GPU memory was 861.75 MB. These results indicate that GAMT-GINE maintains a small model-file footprint and low inference GPU-memory requirements even when a five-model ensemble is used, while supporting rapid batch prediction in a native Windows GPU environment.

## 4. Conclusions

This study proposes GAMT-GINE, a protein–ligand binding affinity prediction model that integrates continuous spatial awareness with multi-task learning. The model adopts a GINE framework without batch normalization and combines basic atomic features with radial basis function distance encoding to represent the three-dimensional geometry of protein–ligand complexes. *K*_d_/*K*_i_ prediction is treated as the primary task, whereas IC_50_ prediction is used as an auxiliary task, enabling the joint utilization of heterogeneous affinity labels. GAMT-GINE achieved competitive predictive performance on both the CASF-2013 and CASF-2016 benchmark test sets. Ablation experiments and feature analyses further demonstrated the importance of continuous distance encoding, the batch-normalization-free design, the multi-task learning mechanism, and the basic atomic features. Data-filtering experiments showed that overly stringent filtering does not necessarily improve external test performance and that maintaining an appropriate balance among data quality, sample size, and structural diversity may be more important. The similarity analysis further indicated that model evaluation results are affected by protein structural similarity between the training and test sets. Further retrospective multi-target candidate prioritization analysis showed that GAMT-GINE outperformed the random-ranking baseline in both high-affinity candidate retention and within-target relative ranking. In the representative PDE10A case, the model exactly recovered the experimentally determined Top-2 candidate set, demonstrating its potential utility for candidate prioritization and candidate-list reduction. Overall, GAMT-GINE achieves a favorable balance among predictive performance, input representation, utilization of heterogeneous affinity labels, and computational efficiency, while exhibiting a certain degree of cross-dataset applicability. Nevertheless, its generalization ability for low-similarity proteins, previously unseen target families, and broader chemical spaces still requires further validation through more rigorous data partitioning and prospective screening experiments. It should be noted that GAMT-GINE predicts equilibrium binding affinity from a given static protein–ligand complex conformation, whereas interaction kinetics require additional information such as *k*_on_, *k*_off_, or residence time. Structure-prediction methods such as AlphaFold 3 and RoseTTAFold All-Atom can generate candidate complex conformations, while GAMT-GINE may serve as a downstream model for affinity scoring and candidate prioritization. Because predicted structures may differ from experimentally resolved structures, this integrated workflow still requires independent validation. Future studies could further incorporate molecular-dynamics conformational ensembles and experimental kinetic data to extend the applicability of GAMT-GINE to previously unseen targets, novel chemical spaces, and dynamic protein–ligand interaction studies.

## Figures and Tables

**Table 1:** Compositions and *pK* statistics of the training and independent test sets.

Dataset	Data Source	Role	Total N	*K*_d_	*K*_i_	IC_50_	*pK* Range	*pK* Median (IQR)
v2016 training set	PDBbind v2016–v2020.R1 overlap	Training for CASF-2013	12,795	4248	3736	4811	0.40–15.22	6.47 (2.64)
v2020.R1 training set	PDBbind v2020.R1	Training for CASF-2016	18,752	6936	4783	7033	0.40–15.22	6.46 (2.60)
CASF-2013	CASF-2013 core set	Independent test	195	65	130	0	2.07–11.52	6.27 (3.38)
CASF-2016	CASF-2016 core set	Independent test	285	107	178	0	2.07–11.82	6.50 (3.12)

**Table 2:** Performance comparisons of different models on CASF-2013.

Model	Training Set	CASF-2013	
		RMSE	*R*_P_
OnionNet	PDBbind v2016	1.503	0.782
SIGN	PDBbind v2016	1.448	0.79
IGN	PDBbind v2020	1.44	0.772
GAMT-GINE (ours)	PDBbind v2016	1.403	0.791

**Table 3:** Performance comparisons of different models on CASF-2016.

Model	Training Set	CASF-2016	
		RMSE	*R*_P_
FAST	PDBbind v2016	1.308	0.810
OnionNet	PDBbind v2016	1.278	0.816
SIGN	PDBbind v2016	1.272	0.816
CurvAGN	PDBbind v2016	1.217	0.831
RF-Score-v3	PDBbind v2016	1.390	0.800
IGN	PDBbind v2020	1.303	0.805
GIGN	PDBbind v2020	1.278	0.810
GAMT-GINE (ours)	PDBbind v2020	1.227	0.831

**Table 4:** Comparisons of GAMT-GINE with emerging biomolecular structure prediction methods.

Aspect	GAMT-GINE	AlphaFold 3	RoseTTAFold All-Atom
Objective	Binding-affinity prediction	Complex structure prediction	All-atom structure prediction and design
Input	Protein–ligand complex structure	Sequences and molecular components	Biopolymer sequences and molecular components
Output	Predicted *pK*_d_/*pK*_i_	Coordinates and confidence estimates	Biomolecular assembly coordinates
Small molecules	Supported as input	Supported	Supported
Affinity	Directly predicted	Not a standard output	Not a standard output
Kinetics	Not predicted	Not predicted	Not predicted
Time-resolved dynamic ensembles	Not generated	Not generated	Not generated
Metrics	*R*_p_, RMSE, and MAE	Ligand RMSD, LDDT/iLDDT, DockQ, and confidence metrics	Structure and pose accuracy
Workflow role	Downstream scoring and ranking	Upstream structure generation	Upstream structure generation

**Table 5:** Experimental and predicted affinities for five PDE10A protein–ligand complexes.

PDB ID	Ligand	Experimental *K*_i_	Experimental *pK*	GAMT-GINE Predicted *pK*	Experimental Rank	Predicted Rank
5C2H	Compound 15 h	8.2 pM	11.086	8.074	1	2
3UI7	C1L	1 nM	9.000	8.121	2	1
3UUO	Compound 1	11 nM	7.959	7.586	3	3
5C28	Compound 6	2.2 μM	5.658	5.039	4	4
4LLX	ZT0434 (compound 18)	1300 μM	2.886	4.238	5	5

**Table 6:** Summary of Node and Edge Features in the Spatial Atomic Graph.

Feature Category	Feature Name	Description
Node feature	Atom type	17-dimensional one-hot encoding
Edge feature	Spatial geometric distance	Continuous 3D spatial cutoff distance (d_ij_ < 5.0 Å)

**Table 7:** Hyperparameters and training settings of GAMT-GINE.

Hyperparameter	Description
Atomic node feature dimension	17
Hidden dimension	128
Number of GNN layers	4
Number of RBF kernels	32
Distance cutoff	5.0 Å
Maximum number of epochs	100
Batch size	16
Optimizer	AdamW
Initial learning rate	1 × 10^−4^
Weight decay	1 × 10^−2^
Dropout rate	0.2
Activation function	GELU
Loss function	Smooth L1
Auxiliary-task loss weight	0.5
Scheduler	ReduceLROnPlateau
Scheduler patience	10 epochs
Learning-rate reduction factor	0.5
Number of folds	5
Checkpoint selection metric	Validation *R*_p_

**Table 8:** Computational resource usage and runtime efficiency of GAMT-GINE on the CASF-2016 test set.

Evaluation Metric	Test Condition	Result
Trainable parameters per model	Single GAMT-GINE model	237,826
Model weight-file size	Single model/five-model ensemble	0.921/4.605 MB
Total preprocessing time from raw structures	285 complexes, 8 CPU worker processes	14.049 ± 0.834 s
Peak aggregate process-tree RSS during preprocessing	Main process and all child processes	4396.81 MB
Model-only inference latency per complex	Batch size = 1, five-model ensemble	14.660 ± 2.152 ms
Model-only inference throughput	Batch size = 32, five-model ensemble	360.340 complexes/s
Total time from graph-file loading to final prediction	285 complexes, five-model ensemble	1.996 ± 0.157 s
Peak CPU RSS during inference	Graph loading, batching, data transfer, and inference	881.54 MB
Peak allocated GPU memory during inference	Five-model ensemble	861.75 MB

The aggregate process-tree RSS was calculated by summing the RSS values of the main process and all child processes and may therefore include duplicated accounting of shared memory pages.

## Data Availability

Publicly available PDBbind and CASF datasets were analyzed in this study and can be accessed at https://www.pdbbind-plus.org.cn/download (accessed on 8 August 2026) and https://www.pdbbind-plus.org.cn/casf (accessed on 8 August 2026), respectively. The source code, data preparation, training and evaluation scripts, dataset split files, pretrained models, similarity experiment checkpoints, derived prediction results, and runtime benchmark outputs generated in this study are publicly available at https://github.com/l2339783088/GAMT-GINE (accessed on 8 August 2026).

## Abbreviations

The following abbreviations are used in this manuscript:

GAMT-GINE	Geometry-Aware Multi-Task Graph Isomorphism Network with Edge Features
*K*_d_	Dissociation constant
*K*_i_	Inhibition constant
IC_50_	Half-maximal inhibitory concentration
*R*_p_	Pearson’s correlation coefficient
RMSE	Root mean square error
MAE	Mean absolute error
RBF	Radial basis function
NMR	Nuclear magnetic resonance
PDB	Protein Data Bank
PLA	Protein–ligand binding affinity
PLI	Protein–ligand interaction
CASF	Comparative Assessment of Scoring Functions
GNN	Graph neural network
SIGN	Structure-Aware Interactive Graph Neural Network
CurvAGN	Curvature-Based Adaptive Graph Neural Network
GIGN	Geometric Interaction Graph Neural Network
GINE	Graph Isomorphism Network with Edge Features
IGN	InteractionGraphNet
RF-Score-v3	Random Forest Score, version 3
3D	Three-dimensional
SD	Standard deviation
TM-score	Template modeling score
GELU	Gaussian error linear unit
RSS	Resident set size
